# Internal and External Factors’ Influence on Recycling: Insights From a Laboratory Experiment With Observed Behavior

**DOI:** 10.3389/fpsyg.2021.699410

**Published:** 2021-07-22

**Authors:** Noah Linder, Sonny Rosenthal, Patrik Sörqvist, Stephan Barthel

**Affiliations:** ^1^Department of Building Engineering, Energy Systems and Sustainability Science, University of Gävle, Gävle, Sweden; ^2^Wee Kim Wee School of Communication and Information, Nanyang Technological University, Singapore, Singapore; ^3^Stockholm Resilience Centre, Stockholm University, Stockholm, Sweden

**Keywords:** environment, behavior, physical context, intention, norms and attitudes

## Abstract

Internal psychological factors, such as intentions and personal norms, are central predictors of pro-environmental behavior in many theoretical models, whereas the influence from external factors such as the physical environment is seldom considered. Even rarer is studying how internal factors interact with the physical context in which decisions take place. In the current study, we addressed the relative influence and interaction of psychological and environmental factors on pro-environmental behavior. A laboratory experiment presented participants (*N* = 399) with a choice to dispatch a used plastic cup in a recycling or general waste bin after participating in a staged “yogurt taste test.” Results showed how the spatial positioning of bins explained more than half of the variance in recycling behavior whilst self-reported recycling intentions were not related to which bin they used. Rinsing cups (to reduce contamination) before recycling, on the other hand, was related to both behavioral intention and external factors. These results show that even seemingly small differences in a choice context can influence how well internal psychological factors predict behavior and how aspects of the physical environment can assist the alignment of behavior and intentions, as well as steering behavior regardless of motivation.

## Introduction

Recent reports stress the need to find more successful ways to encourage environmentally friendly behavior (IPCC, 2014; Brondizio et al., 2019), and behavioral science may assist in the endeavor to understand, predict, and promote pro-environmental behavior (Steg and Vlek, 2009; Clayton et al., 2016; Sörqvist, 2016). The present study expands on Rosenthal and Linder (2021), who documented effects of the external environment on recycling and rinsing behavior. We explore the same phenomena from a more psychological perspective by studying the interaction of intrapsychic determinants and changes in the physical environment. Contemporary environmental psychology covers many studies on how internal psychological factors—e.g., values, norms, and intentions—relate to pro-environmental behavior. Some of the most prominent theoretical models include the theory of planned behavior (TPB) (Ajzen, 1991), the value-belief-norm (VBN) theory (Stern et al., 1999), and the norm activation model (NAM) (Schwartz, 1977). These models generally do a good job of predicting behavior (Montano and Kasprzyk, 2015). Meta-analyses show that the TPB explains around 26–36% of the variance in behavior and 40–49% of intentions (McEachan et al., 2016). Similarly, a study found that personal norm, the main predictor in VBN theory and NAM, accounts for up to 35% of the variance in self-reported recycling behavior (Valle et al., 2005). Research based on attitude-behavior models often describe intention as a key antecedent of behavior. Bamberg and Möser (2007) reinforced this idea in a meta-analysis of psycho-social determinants of pro-environmental behavior, suggesting that intention mediates the impact of other internal variables on behavior. However, scholars have long noted that intentions and behaviors do not always align (Kollmuss and Agyeman, 2002; Rosenthal, 2018). If an objective measure/observation of behavior is used instead of self-reported behavior, the predictability of TPB drops considerably (Armitage and Conner, 2001). In fact, only a minority of studies based on these models measure actual behavior; most studies use self-reports of behavior or intention as criterion variables (Webb and Sheeran, 2006; Sheeran and Webb, 2016). This gap between behavior and its proposed intrapsychic determinants illustrates one of the biggest limitations of the attitude-behavior models, often mentioned in the literature as the value-action gap (e.g., Blake, 1999), the intention-behavior gap (e.g., Sniehotta et al., 2005), or attitude-behavior gap (e.g., Boulstridge and Carrigan, 2000).

The extent to which the characteristics of the physical environment influence pro-environmental behavior has received less attention in contemporary environmental psychology, even though many researchers have called for such studies (Tanner et al., 2004; Steg and Vlek, 2009; Gärling, 2014; Page, 2015; Sörqvist, 2016). A better understanding of how external factors influence behavior might shine a light on some of the unexplained variance in attitude-behavior models. Furthermore, efforts to promote pro-environmental behavior might be more effective if they looked beyond individual motivations. Guagnano et al. (1995) argued that situational factors set boundary conditions for how well attitude-behavior models predict behavior. Features of the physical environment may facilitate or hinder intention leading to action. The physical environment may also shape behavior in the absence of intention, which can help explain some of the intention-behavior gaps commonly found. For example, situational constraints have been shown to explain most of the variation in travel behavior (Klöckner and Blöbaum, 2010), and inconvenient access to recycling infrastructure can cause even the best-intentioned recyclers to not recycle (Guagnano et al., 1995). Even subtle changes in the physical environment, such as whether the light is on or off when someone enters a public bathroom (Dwyer et al., 2015) or smaller serving plates at buffets (Kallbekken and Sælen, 2013), can lead to drastic changes in behavioral outcomes.

How internal and situational factors interact in influencing pro-environmental behavior is even less studied, although researchers have long called for such exploration (Guagnano et al., 1995; Schultz et al., 1995). Recent work has started to address this research gap (Vetter and Kutzner, 2016; Moussaoui et al., 2020; Kaiser, 2021). But only a few studies looked at similar interactions with observed behavior under controlled conditions (Taube et al., 2018; Kaiser and Lange, 2021), and we have found only one such study looking specifically at recycling behavior (Huffman et al., 2014). There have been some studies that looked at how situational factors and internal factors influence or interact with recycling behavior (see e.g., Boldero, 1995; Schultz et al., 1995; Corral-Verdugo, 2003; Barr, 2007; Fumao, 2012); however, they relied mostly on self-reports. In general, recycling research seldom makes direct behavioral observations (Huffman et al., 2014). Such objective measures are needed in the recycling context and to understand pro-environmental behaviors broadly (Steg and Vlek, 2009; Kormos and Gifford, 2014; Lange et al., 2018).

The present study addresses the above-mentioned research gaps by studying the dependencies between self-reported internal factors and the physical environment in which behavioral decisions take place. To this end, we gathered self-reported data on some of the intrapsychic determinants used in explaining recycling behavior. Specifically, we measured personal recycling norms (Schwartz, 1977; Harland et al., 1999), environmental self-identity (Whitmarsh and O’Neill, 2010), biospheric values (Van der Werff et al., 2013), and recycling-related habits (Aarts et al., 1998; Page, 2015; Verplanken, 2018). Finally, we measured the intention to rinse and recycle and observed actual rinsing and recycling behavior.

### Choice Context

This study aimed to explore how self-reported internal predictors compared and interacted with changes in the physical environment. In this mission, we surveyed the participants in connection to a staged “yogurt taste test.” At the end of the taste test, participants had the opportunity to rinse and recycle a used plastic cup that was contaminated with yogurt residue. We manipulated this choice context in two ways. First, in half the conditions, a general waste bin and a recycling bin were placed next to each other and adjacent to the counter and sink where the taste test took place. In the other half, the waste bin was placed further away from the counter, making the recycling bin the nearest option. Second, there was an informational prompt encouraging recycling. One version of the prompt included a visual guide on what constitutes adequate rinsing of contamination from recyclables. Important to note is that recycling contamination is a pressing issue in Singapore, where this study took place, and the local recycling companies will reject even slightly contaminated items. Rosenthal and Linder (2021) reported the effects of these physical manipulations, finding that the use of the recycling bin increased when the waste bin was moved away and the contamination rate was lower when the prompt provided the visual rinsing guide. We add nuance to those findings by addressing the role of intrapsychic factors in these rinsing and recycling behaviors.

First, this design allowed us to compare the effect of the physical manipulations to self-reported internal factors. The effect of the physical manipulation on recycling and rinsing aligned with previous studies. The spatial location of the bins has to do with making recycling more or less convenient; convenience has been shown to heavily influence whether people recycle or not (Domina and Koch, 2002; McKenzie-Mohr, 2011; Osbaldiston and Schott, 2012). And when an option is made to be relatively easier than an alternative, behavior is likely to follow (McKenzie-Mohr and Schultz, 2014). Similarly, prompts, i.e., strategically placed notifications at the point of decision to promote or remind people of a specific behavior (McKenzie-Mohr and Schultz, 2014), have been continuously shown to be effective tools to spur some pro-environmental actions (Durdan et al., 1985; Werner et al., 2009; Tetlow et al., 2014; Moussaoui et al., 2020). As we noted above, even small changes in the physical environment can have a big influence on behaviors. Knowing that people often fail to align their values with actions, we hypothesized that contextual factors, i.e., the location of the bins and the presence of prompts, would be better predictors of recycling and rinsing behavior in the experiment than the self-reported intrapsychic determinants.

Secondly, we explored to what extent the effects of intrapsychic factors depend on features of the choice context. In this mission, we looked at (a) if the relationship between internal determinants of recycling interacted with the different bin placements and, (b) how different prompts interacted with our participants’ rinsing intentions.

To answer these research questions the current research took more of an exploratory approach. This was due to the unique experimental design and ambiguous results from previous research on interactions between intrapersonal and environmental determinants of pro-environmental behavior. For example, to predict how the different placement of the waste bin interacted with the intrapsychic determinants, we noted that Taube et al. (2018) found both environmental attitude and the environment layout explained sustainable travel behavior but found (and even predicted) no interaction between them. And although research has shown that environmental attitudes (an intrapsychic factor) can offset some behavioral costs (an external factor; e.g., Kaiser and Lange, 2021), i.e., an individual could overcome some barriers to recycling, such as inconvenience, because recycling is consistent with their attitudes. In such a situation the “right” behavior (e.g., using the recycling bin) is more difficult to perform. It is unclear if this pattern would emerge when, as in our experiment, the choice context instead makes the “wrong” behavior (using the waste bin) more difficult. In this context, attitude may play a less prominent role and habit may come to the fore. Intuitively, one reason someone would engage in a more difficult “wrong” behavior is because they do so out of habit. In the other choice context of our experiment, the situation is one where the “right” and “wrong” behaviors are equally easy (i.e., when the bins are adjacent to each other). In that situation, we see no obvious reason not to follow, e.g., intention to recycle, and we expect a clear correlation between recycling intention and recycling behavior. On the other hand, when the waste bin is moved a distance away, the “right” behavior might be far more common, and heavily steered by the environmental context without the need to rely on intrapsychic determinants. That is, we predicted that the relationship between the internal variables and recycling behavior is stronger when the bins are adjacent to each other than when the waste bin is moved further away.

Looking at prompts and rinsing behavior, Moussaoui et al. (2020) found that participants who reported higher levels of pro-environmental attitudes performed more energy-saving behavior in the presence of a conservation prompt, however, they did not find statistical support for the interaction between prompts and attitudes (because no one conducted the energy-saving behaviors without the prompt). Similarly, we predicted that the relationship between intrapsychic factors and rinsing behavior is stronger when a rinsing prompt is present.

### Purpose and Hypotheses

In summary, the purpose of the current study was to compare how well internal and external factors predict behavior and study the interactions between intrapsychic factors and features of the choice context. All in all, we tested the following hypotheses:

**Hypothesis 1:** Recycling of the tasting cup will be more strongly predicted by the location of the waste bin than by self-reported intentions to recycle.
**Hypothesis 2:** Rinsing the cup will be more strongly predicted by the presence of a rinsing prompt than by self-reported intention to rinse.
**Hypothesis 3:** The relationship between recycling intention and recycling behavior is stronger when the bins are adjacent to each other than when the waste bin is moved further away.
**Hypothesis 4**: The relationship between rinsing intention and rinsing behavior is stronger when a rinsing prompt is present.

As intentions are the most commonly used antecedent of pro-environmental behavior and have been shown to mediate the impact of other psycho-social variables (Bamberg and Möser, 2007), we started with analyzing this internal variable, but we also test and controled for the other internal variables measured.

## Materials and Methods

### Sample Size and Participants

This study used the same sample as in Rosenthal and Linder (2021). Participants were sampled from a list of roughly 19,000 email addresses of undergraduate students at Nanyang Technological University, Singapore. An email recruitment letter was sent to a random sample of 4,000 of those email addresses. The recruitment letter invited the students to participate in a “yogurt taste test.” After 2 days, individuals who had not already signed up received a reminder. After signing up, the participants selected an experimental session they wished to attend. There were 63 sessions over two consecutive weeks in March and April 2019, which were rotated to remove confounding with the day of the week and time of the day the sessions took place. There were up to 9 participants in each session. Not all the sessions were full either due to unpopular time slots or last-minute drop-outs. Everyone who received invitations had the option to self-schedule into experimental sessions up until they ran in an effort to maximize enrollment. In total 409 participants signed up for a session and participated in the study. They were 61% female and ranged in age from 18 to 28 years (*Mdn* = 22 years).

### Procedure

After arriving for their study sessions, participants received a small plastic cup containing a sample of a yogurt drink. They were then directed into a private tasting booth by a researcher who gave a scripted explanation of the taste-test procedure and prompted them to dispatch the cup when returning from the tasting booth. After receiving the instructions, they entered a private tasting booth out of view of the researchers and other study participants. The booth included a tasting station at a small sink, a recycling bin, and a general waste bin. A recycling prompt was affixed to the wall above the recycling bin.

Participants tasted the yogurt drink, completed a short survey to share their thoughts about it, and disposed of the recyclable plastic cup in one of the bins before exiting the tasting booth. During the experiment, participants were unaware that their recycling behavior was being observed, believing they were participating only in a taste test study.

Following the taste test, participants completed an online survey on their personal smartphone or a tablet the researchers provided and collected an incentive of 10 Singapore dollars. A debriefing statement at the end of the study revealed the true focus on recycling behavior and requested their consent to use their recycling data in our analyses. Three participants declined that consent, and two participants took their cups with them when they left the study. We excluded the data from those five participants. There were five additional participants, in the same group, whom we believe overheard earlier participants speaking loudly about the recycling-focused questions. We also excluded those participants out of concern their rinsing and recycling behaviors were not spontaneous.

### Self-Report Measures

To measure internal factors, we asked participants to answer an online survey after the taste test. We used items from De Groot and Steg (2008) to measure biospheric, altruistic, and egoistic value orientations. Participants responded on five-point scales that ranged from 1 (*not at all important*) to 5 (*extremely important*), indicating to what extent the items reflected guiding principles in their lives. For the current study, we focused on the measurement of biospheric values, which included “preventing pollution,” “respecting the earth,” “unity with nature,” and “protecting the environment.”

We measured environmental self-identity using items from Van der Werff et al. (2013). Participants indicated on a five-point scale ranging from 1 (*strongly disagree*) to 5 (*strongly agree*) their agreement with the statements, “Acting environmentally friendly is an important part of who I am,” “I am the type of person who acts environmentally friendly,” and “I see myself as an environmentally friendly person.

We assessed recycling personal norm based on Harland et al. (1999). Participants indicated their agreement with the statements, “I feel a strong personal obligation to recycle,” “I am willing to put extra effort into recycling,” and “I would feel guilty if I didn’t recycle.” Response options ranged from 1 (*strongly disagree*) to 5 (*strongly agree*).

We measured behavioral intention using items adapted from prior research (e.g., Malek et al., 2017). Participants indicated their agreement that in the following month they “expect to,” “plan to,” and “will try to” recycle/rinse.

Lastly, we measured habit strength for recycling and rinsing using a shortened version of the self-report index of habit strength scale (Verplanken and Orbell, 2003; Honkanen et al., 2005). Participants indicated their agreement that recycling and rinsing recyclables are things “I do frequently,” “I do without having to consciously remember,” “I feel weird if I don’t do,” and “I don’t have to think about doing.”

### The Physical Context

**FIGURE 1:**
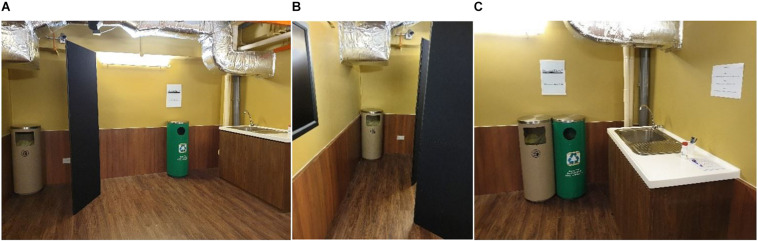
Manipulating the spatial positioning of the bins. The taste test booth with the recycling bin and general waste bin spatially separated (**panel A**) and adjacent to each other (**panel C**). In the Far condition, the general waste bin is moved further away but clearly visible upon entering the taste test booth (**panel B**), figure modified from Rosenthal and Linder (2021).

**FIGURE 2:**
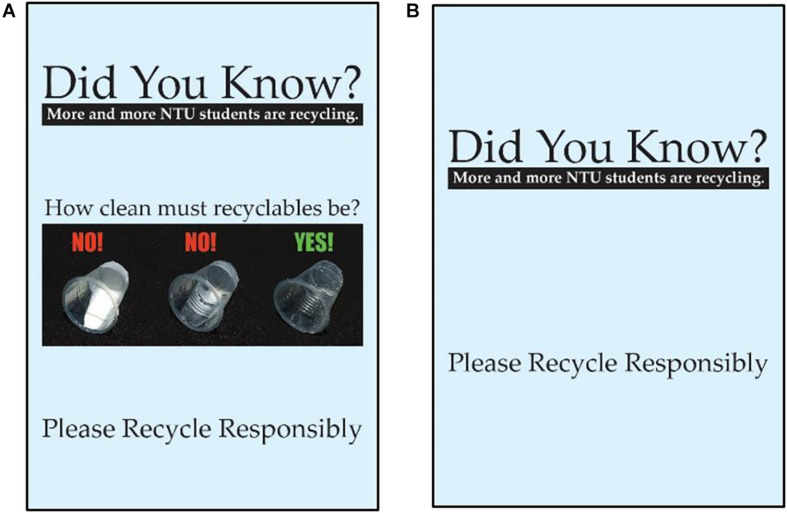
Prompts used in the study. **(A)** The visual guide to rinsing showing how clean a recyclable have to be in order to avoid risking contamination, **(B)** a “control prompt” with no information about rinsing (published with permission by Authors).

The experiment comprised a between-participant 2 × 2 factorial design. In the “*near*” condition, the recycling bin and general waste bin were located next to the counter in the tasting booth and adjacent to each other. In the “*far*” condition, the waste bin was moved roughly four meters away to the entrance of the tasting booth (see Figure 1), while the recycling bin remained in the same location. Located on the wall, above the recycling bin was an informational prompt asking the participants to recycle responsibly. There were four versions of the prompt with different information about rinsing recyclables. Two of the versions included a visual guide showing how clean a cup must be to be recycled (see Figure 2). Those versions of the prompt were more effective at reducing contamination than the two versions that did not include the visual guide (Rosenthal and Linder, 2021). Since the current study is partly interested in external factors that influence rinsing, our analysis of rinsing behavior uses data only from participants who saw either the visual rinsing guides or a control prompt without any rinsing information. This allows us to compare the effects of intrapsychic factors with the effect of an external factor we already know works.

### Behavioral Measurements

Data on recycling and rinsing behavior were collected by examining the taste test cups after each experimental session. Each cup had a unique hidden marking that allowed us to associate it with individual survey responses. For each cup, we indicated whether it was in the recycling bin or general waste bin and if it was rinsed or not rinsed. These dependent variables were coded with values of 0 (*general waste bin*; *not rinsed*) and 1 (*recycling bin*; *rinsed*). To assess inter-rater reliability when determining if the cup was rinsed (or not) two of the authors and a graduate research assistant separately evaluated the contamination level of 50 cups from the first day of the experiment. We assessed interrater reliability using Krippendorff’s alpha (Hayes and Krippendorff, 2007) and found good reliability (α = 0.92, [95% CI: 0.88, 0.96]).

### Attention Check

Since the prompts require cognitive processing to be effective, we let the participants indicate their level of agreement with the statement: “At the taste test area, there was a poster encouraging recycling.” Most participants agreed (36%) or strongly agreed (33%) of seeing the prompt. Some participants neither agreed nor disagreed (15%), and a few disagreed (11%), or strongly disagreed (5%). We decided to retain all participants in our sample for greater external validity; in the real world, not everyone pays attention to informational prompts.

## Results

### Individual Differences in Predictor Variables

The descriptive statistics and zero-order correlation matrix for all variables are presented in Table 1. Most scales had acceptable or better reliability according to general rules of thumb (Cronbach’s α > 0.9 is excellent, >0.8 is good, and >0.7 is acceptable; Gliem and Gliem, 2003), except for the “hedonic” and “altruistic” value scales, these were excluded from further analysis.

**TABLE 1:** Means, standard deviation, Cronbach’s alpha, and Pearson correlation matrix for all variables (*N* = 399).

**Internal factors**	***Mean***	***SD***	**α**	***1***	***2***	***3***	***4***	***5***	***6***	***7***	***8***	***9***	***10***	***11***	***12***	***13***	***14***
1. Biospheric values	3.73	0.76	0.85	1													
2. Altruistic values	4.01	0.57	0.66	0.54***	1												
3. Egoistic values	3,23	0.61	0.71	0.27***	0.26***	1											
4. Hedonic values	3.89	0.57	0.68	0.29***	0.24***	0.43**	1										
5. Environmental SI	3.42	0.85	0.85	0.58***	0.25***	0.16**	0.14**	1									
6. Recycling personal norm	3.57	0.82	0.78	0.51***	0.26***	0.12*	0.10*	0.69***	1								
7. Intention to recycle	4.02	0.76	0.87	0.41***	0.25***	0.08	0.09	0.50***	0.61***	1							
8. Intention to rinse	3.74	1.02	0.90	0.26***	0.22***	–0.04	–0.02	0.27***	0.32***	0.40***	1						
9. Recycling habit	3.11	0.96	0.88	0.35***	0.09	0.07	0.07	0.56***	0.65***	0.59***	0.27***	1					
10. Rinsing habit	2.62	1.15	0.92	0.15**	0.03	0.04	0.00	0.26***	0.27***	0.27***	0.58***	0.36***	1				
**External factors**																	
11. Waste bin near/far	–	–	–	–0.05	–0.04	–0.09	–0.01	0.07	0.07	0.04	0.12*	0.06	–0.10	1			
12. Prompt with/without	–	–	–	0.03	0.06	0.03	0.06	–0.07	–0.06	–0.01	0.01	–0.05	–0.03	0.04	1		
**Outcome variables**																	
13. Choice of bin	–	–	–	–0.02	–0.03	–0.06	–0.00	0.04	0.09	0.05	0.00	0.4	–0.09	0.05	0.67***	1	
14. Rinsed yes/no	–	–	–	0.03	–0.01	0.09	–0.01	0.03	0.08	0.07	0.18***	0.13*	0.14**	0.12*	0.14**	0.26***	1

*p < 0.05, **p < 0.01, ***p < 0.001, α = Cronbach’s alpha.

### Behavioral Measures

In the final sample, 399 plastic cups were collected from the two bins, 196 when the bins were co-located, in the *”near*” condition and 206 in the *“far”* condition where the waste bin was moved away from the recycling bin. In the near condition 30% (*n* = 59) of participants used the recycling bin and 70% (*n* = 137) used the general waste bin. In the far condition, 95% (*n* = 193) of the cups were found in the recycling bin and only 6% (*n* = 10) in the general waste bin. Overall, 63% (252) of the participants used the recycling bin. Out of all cups, 18% (*n* = 70) were rinsed. This included 25% (*n* = 63) of the cups in the recycling bin and 5% (*n* = 7) in the general waste bin. When the rinsing prompt was present 23% (*n* = 47) of the cups were rinsed, when participants were exposed to the control prompt on the other hand, 14% (*n* = 14) rinsed their cups.

### Hypothesis Testing

#### Comparing Internal and External Predictors

We tested Hypothesis 1 by predicting recycling behavior with the bin location manipulation and recycling intention. Since recycling behavior was a binary variable (i.e., participants used one bin or the other), we used binary logistic regression to predict which bin participants used to dispose of the tasting cup. We also included recycling habit as a control variable. We wanted to control for habits because habits have been shown to sometimes moderate the attitude-behavior relationship of recycling behaviors (Aarts et al., 1998; Page, 2015; Verplanken, 2018). Furthermore, researchers have argued that after a habit is established the behavior should no longer be considered to be goal-directed (Miller et al., 2019).

The model explained between 40% (Cox & Snell R-square) and 55% (Nagelkerke R-square) of the variance in recycling behavior. Recycling behavior was unrelated to intention (OR = 1.12 [95% CI: 0.71, 1.76], *p* = 0.623) and habits (OR = 0,98 [95% CI: 0.68, 1.40] *p* = 0.895) but significantly related to the bin location manipulation (OR = 44.783 [95% CI: 22.11, 90.71], *p* < 0.001). In this case, the behavior was heavily dominated by the external factor, which supports Hypothesis 1 (Table 2).

**TABLE 2:** Logistic regression: choice of bin as dependent variable, recycling intention, bin manipulation and recycling habits as independent variables.

		**95% CI for Odds ratio**		
	**B(SE)**	**Odds Ratio**	**Lower**	**Upper**
Constant	−1.22(0.76)			
Recycling intention	0.11 (0.36)	1.12	0.71	1.76
Waste bin near/far	3.80***(0.36)	44.78	22.11	90.71
Recycling habits	−0.02(0.18)	0.98	0.68	1.34

R^2^ = 0.40 (Cox & Snell), 0.55 (Nagelkerke), ***p < 0.001.

We re-estimated this model using the other internal factors—viz. biospheric values, environmental self-identity, personal recycling norm—as the independent variable in place of recycling intention. We removed habits from the model due to multicollinearity concerns, as there was a strong correlation between habits and the other internal variables. We also re-ran the original model without controlling for habits. The result of the new models was very similar to the first one—none of the internal factors significantly predicted recycling behavior (See Table 3).

**TABLE 3:** Logistic regressions, with alternative predictor variables.

		**95% CI for Odds ratio**		
	**B(SE)**	**Odds Ratio**	**Lower**	**Upper**
**Retest of hypothesis 1 (*N* = 399)**				
Biospheric values	0.08 (0.19)	1.09	0.75	1.57
Environmental self-identity	−0.03(0.17)	0.97	0.70	1.34
Personal recycling norm	0.17 (0.17)	1.18	0.84	1.66
Recycling intention	0.95 (0.19)	1.10	0.77	1.58
**Retest of hypothesis 2 (*N* = 303)**				
Biospheric values	0.02 (0.20)	1.02	0.68	1.51
Environmental self-identity	0.05 (0.18)	1.05	0.74	1.45
Personal recycling norm	0.14 (0.18)	1.15	0.80	1.64
Rinsing intention	0.61(0.18)**	1.84	1.28	2.64

**p < 0.01.

We took a similar analytical approach to test Hypothesis 2, using the subsample of participants who saw either a rinsing prompt with the visual rinsing guide or the control prompt without any mentions of rinsing (*n* = 303). This model predicted rinsing behavior as the dependent variable, with the prompt and rinsing intention as independent variables, again, controlling for rinsing habit and whether participants used the waste bin or recycling bin. The reason to control for bin choice is that there is no benefit of rinsing cups that ended up in the waste bin (Table 4). This model explained between 16% (Cox & Snell R-square) and 25% (Nagelkerke R-square) of the variance in rinsing behavior. Rinsing behavior was significantly related to both the intention to rinse (OR = 1.57 [95% CI: 1.04, 2.40], *p* = 0.034) and the presence of the rinsing prompt (OR = 2.01 [95% CI: 1.02, 4.30], *p* = 0.044). It was also related to bin choice (OR = 10.15 [95% CI: 3.84, 26.84], *p* = 0.001), but unrelated to rinsing habit (OR = 1.25 [95% CI: 0.91, 1.74], *p* = 0.175). These results suggest both internal and external factors predicted rinsing behavior.

**TABLE 4:** Logistic regression: rinsing as dependent variable, rinising intention, habit, and choice of bin as independent variables.

		**95% CI for Odds ratio**		
	**B(SE)**	**Odds Ratio**	**Lower**	**Upper**
Constant	−6.01(0.96)			
Intention to rinse	0.45*(0.21)	1.57	1.04	2.40
Rinse prompt	0.74*(0.37)	2.01	1.02	4.30
Rinsing habit	0.23 (0.17)	1.25	0.91	1.74
Choice of bin	2.32**(0.50)	10.15	3.84	26.84

R^2^ = 0.16 (Cox & Snell), 0.25 (Nagelkerke), *p < 0.05, **p < 0.01.

Again we re-estimated the model (excluding habit) using the other internal factors as the independent variable in the place of rinsing intention. Notably, none of the other internal factors significantly predicted rinsing behavior (Table 3).

#### Internal Factors Conditioned on External Factors

Next, we explored how internal factors interacted with the physical manipulations. Our third hypothesis predicted that the relationship between recycling intention and choice of bin would be weaker when the waste bin was moved a distance away. Using a bivariate Pearson correlation we tested how well intention to recycle predicted observed recycling behavior in the *near* condition (*r* = 0.07, *p* = 0.316, *df* = 194) and in the *far* condition (r = −0.06, *p* = 0.350 *df* = 201) alone. These results indicate that recycling intention failed to predict recycling behavior whether the waste bin was near or far. We extended this analysis by adding an interaction effect to our earlier binary logistic regression (Table 2, no longer controlling for habit). This modeled the effect of recycling intention conditioned on the bin location manipulation. Failing to support Hypothesis 3, the interaction effect was not significant (OR = 0.51 [95% CI: 0.17, 1.48], *p* = 0.213), note that interaction models might need up to 16 times the sample size to estimate an interaction compared to a model that estimates main effects (see for example; Gelman, 2018) and a potentially under-powered model could explain this null-findings (we explore this further below; see sensitivity analysis).

Finally, we tested whether the relationship between rinsing intention and rinsing behavior is stronger when there was a visual guide to rinse on the prompt (Hypothesis 4). Again, we needed to control for what bin they choose when looking at effects from the rinsing prompt. Hence, we used a binary logistic regression and looked at how well intention to rinse predicted rinsing behavior without a visual guide to rinse on the prompt (*n* = 100), controlling for choice of bin. In this scenario, intention to rinse was not significantly related to rinsing behavior (OR = 1.71 [95% CI: 0.89, 3.28], *p* = 0.108; Table 5). This suggests that when there was no rinsing prompt, intention to rinse was a poor predictor of behavior. However, considering the uneven distribution in our dependent variable in this choice context, i.e., only 14 participants rinsed their cup (without a visual guide to rinsing on the prompt), this model is also at risk of being underpowered (we address these power considerations under caveats and limitations).

**TABLE 5:** Results of the logistic regression, rinsing as dependent variable, rinsing intention and choice of bin as independent variables.

		**95% CI for Odds ratio**		
	**B(SE)**	**Odds Ratio**	**Lower**	**Upper**
**No rinsing prompt (*N* = 100)**				
Constant	−4.62(1.47)			
Intention to rinse	0.54 (0.33)	1.71	0.89	3.28
Choice of bin	0.94 (0.70)	2.56	0.65	10.09
*R*^2^ = 0.05 (Cox & Snell), 0.09 (Nagelkerke)				
**Rinsing prompt (*N* = 203)**				
Constant	−6.26(1.20)			
Intention to rinse	0.67(0.23)**	1.96	1.25	3.07
Choice of bin	3.05(0.75)***	21.07	4.87	91.232

R^2^ = 0.20 (Cox & Snell), 0.30 (Nagelkerke), **p < 0.01, ***p < 0.001.

In contrast, when the visual guide to rinsing was present on the prompt (N = 203), intention was significantly related to rinsing (OR = 1.96 [95% CI: 1.25, 3.07], *p* = 0.003; Table 3). This finding is consistent with Hypothesis 4. Again we extended this analysis by adding an interaction effect. We extended our binary logistic regression model (Table 4) to include the effect of rinsing intention conditioned on the prompt information manipulation, again, the interaction effect was not significant (OR = 1.13[95% CI: 0.52, 2.45], *p* = 0.759). Thus, we observed no conclusive evidence for the relationship between rinsing intentions and rinsing behavior to depend on the presence of a rinsing prompt.

### Sensitivity Analysis

To explore the null findings of the interaction models, we used G^∗^Power 3.1.9.7 to conduct a two-tail *post hoc* sensitivity analysis of the interaction effects predicting each dependent variable. We report the minimum OR detectable at 80, 90, and 95% power for both effect directions, i.e., OR < 1 and OR > 1. For both analyses, the alpha error probability was 5% and there was a 5% probability of recycling/rinsing assuming the null hypothesis. For the prediction of recycling, the sample size was 399 and we used a value of 50% for the R-square associated with the main effects. The high value is mainly because the manipulation of the bin location had a very large effect on recycling behavior. We then estimated the critical OR for the interaction effect at 80% (OR_crit_ = 0.43, 2.33), 90% (OR_crit_ = 0.38, 2.61), and 95% (OR_crit_ = 0.35, 2.86). For the prediction of rinsing, the sample size was 303 and we used a value of 30% for the R2 associated with the main effects. We then estimated the critical OR for the interaction effect at 80% (OR_crit_ = 0.44, 2.28), 90% (OR_crit_ = 0.39, 2.55), and 95% (OR_crit_ = 0.36, 2.78). Neither of our observed interaction effects meets the critical OR at any of the specified power levels, indicating that we would need to observe substantially larger effects to have adequate power given our sample sizes.

## Discussion

This study explored how self-reported intrapsychic determinants and the physical environment interacted and predicted observed recycling behavior and rinsing. We found no relation between recycling the cup and internal factors in any choice context; instead, the decision to recycle was heavily determined by situational factors. In contrast, rinsing behavior was related to both behavioral intention and external factors. We found the effect of rinsing intention to be significant only when the context promoted rinsing (with the prompt displaying a visual guide to rinsing), but the results provide no definite evidence that rinsing intention depended on the prompt to align with behavior. We briefly address some null findings and discuss these findings in relation to the two types of behaviors below.

### Choosing to Recycle (or Not)

Contrary to our predictions, even when participants were in a seemingly optimal situation to act on their intentions with easy access to both bins (in the *near* condition) we found no relationship between self-reported intentions (or any other internal variable) and observed recycling behavior. Only when the general waste bin was moved away, so that the recycling bin was relatively closer at hand, did our participants with high self-reported intentions consistently follow through and throw their cup in the “green” bin. However, so did most participants regardless of intention, making intention a poor predictor of recycling behavior in our study. Note that even though the intention to recycle was generally high amongst our participants (*M* = 4.02, out of 5), only 35% of participants chose to use the recycling bin when they had easy access to both bins. These findings align with the commonly found intention-behavior gap (Kollmuss and Agyeman, 2002).

Since intended behaviors are goal-directed, a plausible explanation for this null finding is that some participants failed to recycle simply because they had other goals in mind after conducting the taste test; i.e., the reason for not acting on an existing green self-identity could be because another goal-directed behavior was prioritized (e.g., finishing the task as fast as possible to make room for the next participant). A common reason to why people fail to act on intentions is that they have different goals, priorities, and conflicting intentions that are more prevalent in certain decision situations (Conner et al., 2016). Such conflict in goals could explain why some participants nevertheless dispatch their cup in the general waste bin, despite general intentions to act pro-environmentally. Furthermore, in the study we used an operationalizations of intention that are typically used in related research, i.e., “following month they *expect to, plan to, and will try to* recycle/rinse.” However, such measure might give an indication of a more “general intention” that is somewhat detached from this specific decision context. Another operationalization of intention that better captures intended behavior in certain situations might generate different results. More research is needed to explore such differences in operationalization.

Our results align with previous research showing how the spatial layout and convenience can have a big impact on recycling behavior, often overriding intrinsic motivation, e.g., how inconvenient access to recycling facilities can be a stronger predictor of actual behavior than intrapsychic determinants (Guagnano et al., 1995). Showcasing how external factors can steer behavior without the need to refer to intentions. Our results go even further than previous studies, indicating how even seemingly small differences in convince can override behavioral intentions and have a big impact on recycling behavior. Considering that having to rinse the cup before recycling means more effort than just throwing the cup away in the general waste bin, it is likely that some participants perceived recycling to be less convenient even in the adjacent condition, thus convenience could help to explain the seemingly low recycling rate in the *near* condition as well as the big discrepancies between the two conditions. It is important to note that contextual factors could be especially potent in unfamiliar environments where actions are not guided as much by past behaviors and habits (Verplanken, 2018), and it is also likely that participating in an experiment could increase attention to the context (we address this further below, see potential caveats and limitations).

### Determinants of Rinsing

With rinsing behavior, on the other hand, our results indicate that both the visual guide to rinsing on the prompt and intention to rinse were significantly related to lower contamination rates of the cups. However, no other internal factor—viz. biospheric values, environmental self-identity, personal recycling norm—was related to rinsing. And at a closer look, we only found a significant relationship between intention and behavior when the rinsing prompt was present. Note, however, that the null finding for the relationship between intention and behavior without the prompt could stem from an underpowered model (because so few participants choose to rinse in that scenario), and failing to see a significant interaction effect we cannot conclusively say that intention to rinse was conditioned on the prompt. When the prompt was present, rinsing rates increased from 14 to 23%, and as reported in Rosenthal and Linder (2021) the prompt significantly lowered contamination rates. A plausible explanation for why the rinsing prompt was successful in encouraging rinsing and might have promoted the alignment of intentions and action is that it could have primed pro-environmental values/attitudes/norms and activated a goal-directed behavior that aligned with them, i.e., well-intended participants might be more likely to act on their intentions when they are reminded of them by the prompt. This explanation is consistent with Moussaoui et al. (2020) study that showed how pro-environmental attitudes increased the likelihood of sustainable action in the presence of a prompt. Our study shows further similarities to the Moussaoui et al. (2020) in that we also failed to find evidence of an interaction between our internal factors and the prompt. More research is needed to untangle these somewhat ambiguous findings.

Overall it seemed that intention was a better predictor for rinsing than recycling at our staged taste-test. This is aligning with previous studies that show how intention can be a better predictor for new and less established behaviors (Honkanen et al., 2005) in comparison with more routine behaviors, which are often mediated by habit strength (Verplanken, 2018). Contamination of recyclables was an issue in Singapore that had only recently been highlighted at the time of the data collection, and the need to rinse recyclables was both less known and a less established behavior than recycling. The current results are consistent with the idea that intention is a stronger predictor for more novel or rare behaviors. To test this possible explanation, we conducted a *post hoc* paired *t*-test comparing means of habit strength for recycling and rinsing. Results showed a significantly stronger recycling habit (*M* = 3.11, *SD* = 0.96) compared to rinsing habit (*M* = 2.62, *SD* = 1.14), *t*(402) = 8.06, *p* < 0.001, giving it some support. This adds another layer of complexity to how the physical environment interacts with people’s intentions. Some relations between intention and behavior might be more heavily determined by contextual factors than others, especially as more established, habitual behavior often is automatically activated by contextual cues which can be powerful drivers of day-to-day behaviors and hard to break free from, even overriding intrinsic motivation to change (Neal et al., 2011). We encourage future research efforts to better understand how different types of behavior interact with external factors. Identifying when environmental factors are especially important for the alignment of intentions and behavior could lead to a better understanding of when people act (or don’t act) on their pro-environmental values as well as providing insights on how to design environments that encourage sustainable actions.

## Addressing Potential Caveats and Limitations

It is important to note some potential caveats of this study. Because participants were invited to participate in a “yogurt taste test” and were unaware of the true focus of the study (recycling behavior) they received the survey and reported (e.g., intention to recycle) after they finished the yogurt-tasting and threw away their cups. This was a conscious decision to limit the risk of participants figuring out the true purpose of the experiment and bias our dependent variables of recycling behaviors. However, this meant that their choice of actions could have influenced their subsequent answers in the survey. Important to note though is that they answered the survey before debriefing, and were still unaware of the true focus of the study. For rinsing behavior, however, this possibility increases when we see a significant relationship between behavior and intention. Since we used prompts to get people to rinse recyclables, it is possible that some participants started rinsing (for the first time) during our experiment and then intended to keep doing it. It is therefore likely that the significant relationship between rinsing intention and behavior is somewhat inflated. However, there was no significant difference in intention to rinse between the two groups with and without prompt, respectively, and mean intention to rinse was about the same in the control group (*M* = 3.70) compared to the participants in the rinsing prompt condition (*M* = 3.72). Nevertheless, this potential bias should be kept in mind when interpreting the results.

As for all laboratory experiments, it is also important to reflect on the ecological validity of these findings. The procedure of the experiment is likely to have some influence on behavior. For one, participants found themselves in an atypical situation (i.e., a taste test in a university research lab), and their behavior might not reflect how they would act in a known environment (e.g., at home). In this instance, the laboratory setting may have disrupted the normal mental process where internal factors would have more impact on behavior, whereas other factors such as, e.g., social desirability could have a relatively large impact in the experimental situation. As a result, participants may have been unduly affected by the situation. However, it is very likely that some of the above-mentioned confounds also exist outside the laboratory; people will often be distracted by other tasks, or goals, when choosing to recycle (or not), and not being aware of the possibility of recycling, or the general waste bin, even though it is close to hand, might be a true effect of how context influences behavior (especially in new environments). Considering the somewhat optimal situation to recycle that our participants found themselves in, and still didn’t do so, we consider it likely that the attitude-behavior gap we observed could exists also outside the laboratory.

Lastly, we want to mention our sample size and power in regards to the null findings. Our sample size for the binary logistic regression models looking at main effects are mostly above the general rule of thumb with an Events Per Variable (EPV) of ≥10, i.e., the number of observations in the smaller of the two outcome groups relative to the number of variables is equal to, or above 10 (van Smeden et al., 2019). However, as noted in the Results section, the model on rinsing behavior that failed to see a significant effect was at risk of being underpowered according to this rule, with an EPV of 7 (see the first model in Table 4). Consensus is lacking on the right criteria to determine what sample size should count as adequate for binary logistic prediction (van Smeden et al., 2019). Some have argued that the EPV ≥ 10 rule is sometimes too strict (Pavlou et al., 2016) whilst others argue for an even higher threshold in general (Ogundimu et al., 2016). Either way, we want to highlight, again, that a potentially underpowered model could explain the null finding between intention to rinse and behavior in the scenario without the visual guide to rinsing on the prompt. The main power concern we have is in regards to the interaction models. To address this caveat we ran a *post hoc* sensitivity analysis, the results indicated that we would need to observe larger effects to have adequate power given our sample sizes. And considering that interaction models often need a much higher cell number than testing for a main effect (Gelman, 2018) we can’t rule out that any of these null results are due to a lack of power. We welcome future studies that aim to replicate these findings as well as studying similar interactions with a bigger sample.

## Implications

Our results highlight the importance of measuring actual behavior and that relying on measurements of intentions or self-reports as criterion variables for behavior risk producing weak conclusions. The results present novel insights on how even seemingly small differences in a choice context greatly influence how well intention (and other intrapsychic determinants) predicts behavior, and how aspects of the physical environment might assist the alignment of behavior and intentions, as well as steering behavior regardless of motivation. Models that incorporate the physical context into their explanations of decision-making are gaining popularity. Two such models are the comprehensive action determination model (CADM; Klöckner and Blöbaum, 2010), and the integrated model of behavioral prediction (IMPB) (Kasprzyk et al., 1998). These models have shown some additional success in predicting behavior (e.g., Klöckner and Oppedal, 2011), but are still far from being considered leading accounts of pro-environmental behavior. Our results support the use of such holistic models that better account for things like situational factors and habits.

Environmental campaigns and interventions often strive to foster intentions to act through information persuasion messages which is consistent with theories that emphasize rezoned, deliberative and reflective processes. Unfortunately, persuasion efforts to change people’s behaviors with information campaigns alone are often ineffective approaches (McKenzie-Mohr, 2011). The very popular attitude-behavior models might struggle as frameworks for behavior change because they imply that to change a behavior, first beliefs, values, norms, and attitudes must be changed. Not surprisingly, such deep-seated traits are often heavily defended by both conscious and unconscious psychological mechanisms. And even if the campaigns would manage to change attitudes and intentions among groups to behave more pro-environmentally, their actual behaviors might be more forcibly shaped by other factors in the end. As our experiment indicated, even small changes in the physical environment can steer behavior without the need to rely on intention or values. This gives valuable insights into the extent that characteristics of the physical environment can influence pro-environmental decisions. As society struggles to transform to reach the Paris agreement and other environmental targets, and large scale behavior changes get increasingly urgent, our study supports previous ones that call for more attention to aspects of the physical environments’ effect on pro-environmental behavior (Tanner et al., 2004; Steg and Vlek, 2009; Gärling, 2014; Page, 2015; Sörqvist, 2016; Kaaronen and Strelkovskii, 2020). Thought-through design of the physical landscapes could help facilitate people’s intentions by affording the possibility to act pro-environmentally and reduce the intention-behavior gap (Kaaronen, 2017), as well as getting people to act without the need of relying on intention. Furthermore, because intended behavior is goal-directed and goals constantly switch throughout the day, a more static environmental context that supports pro-environmental choices could lead to reoccurring sustainable actions even when other goals are in mind.

We want to be clear here that we are not trying to downplay the importance of environmental values and intentions in society as they may very well be a prerequisite for any substantial sustainability transformation. For example, the values and attitudes of decision-makers, policymakers, and planners play a crucial role in governance and design processes of, e.g., laws and infrastructures that encourage sustainable actions. Instead, we aim to highlight the potential of steering pro-environmental behavior through manipulation of the physical environment and see it as a currently underutilized complement to conventional approaches and narratives. We end by noting that more studies are needed to confirm any policy relevance of the current findings. However, our findings indicate that an unexpected research potential lies in combining the field of climate action and transformation in cities with the research frontier ignited herein, as the sum of fossil fuel-related behaviors in urban areas aggregate to a disproportionately large driver of global environmental change (Grimm et al., 2008; Colding et al., 2019). More knowledge on how, e.g., the urban form and infrastructures affect pro-environmental behavior could prove to be particularly relevant (Kaaronen and Strelkovskii, 2020).

## Data Availability Statement

The study data is uploaded to OSF and available at: https://osf.io/dbwj7/?view_only=ccf69aa8c7b8411a89754d0bf2afaa8e.

## Ethics Statement

This study was approved by the Institutional Review Board at Nanyang Technological University. Written informed consent was obtained from all participants for their participation in this study. The participants signed two consent forms, one before the experiment and once again after being informed of the true purpose of the study.

## Author Contributions

NL: conceptualization, methodological design, collection of data, analyzing data, and writing manuscript. SR: conceptualization, project administration, methodological design, data analysis, and writing. PS: data analyzing, conceptualization, and writing. SB: conceptualization and writing. All authors contributed to the article and approved the submitted version.

## Conflict of Interest

The authors declare that the research was conducted in the absence of any commercial or financial relationships that could be construed as a potential conflict of interest.
